# Risk factors and strategies for relapse prevention among individuals living with serious mental illness in South Africa: Qualitative inquiry from patients’ and caregivers’ perspectives

**DOI:** 10.1371/journal.pone.0309238

**Published:** 2024-08-22

**Authors:** Joyce Protas Mlay, Thirusha Naidu, Suvira Ramlall, Sbusisiwe Sandra Mhlungu, Makhosazane Zondi, Richard Lessells, Jennifer I. Manuel, Andrew Tomita

**Affiliations:** 1 Discipline of Public Health Medicine, School of Nursing and Public Health, University of KwaZulu-Natal, Durban, South Africa; 2 Health Economics and HIV and AIDS Research Division (HEARD), University of KwaZulu-Natal, Durban, South Africa; 3 Discipline of Behavioural Medicine, School of Nursing and Public Health, University of KwaZulu-Natal, Durban, South Africa; 4 Discipline of Psychiatry, Nelson R Mandela School of Medicine, University of KwaZulu-Natal, Durban, South Africa; 5 Private Practice, Durban, South Africa; 6 Centre for the AIDS Programme of Research in South Africa (CAPRISA), Durban, South Africa; 7 Discipline of Psychology, School of Applied Human Sciences, Pietermaritzburg, South Africa; 8 KwaZulu-Natal Research Innovation and Sequencing Platform (KRISP), College of Health Sciences, University of KwaZulu-Natal, Durban, South Africa; 9 School of Social Work, University of Connecticut, Hartford, Connecticut, United States of America; 10 Centre for Rural Health, School of Nursing and Public Health, University of KwaZulu-Natal, Durban, South Africa; NYU Grossman School of Medicine: New York University School of Medicine, UNITED STATES OF AMERICA

## Abstract

**Introduction:**

Relapse among individuals with serious mental illnesses in resource-limited contexts, including South Africa, is a significant concern. To date, the risks for relapse among this population is well documented, but little is known about prevention strategies to reduce its occurrence in these resource-limited settings. Therefore, this qualitative study explores the risk factors and strategies for relapse prevention from the patients’ and caregivers’ perspectives.

**Methods:**

We conducted audio-recorded face-to-face in-depth interviews to capture the lived experience of relapse of inpatient study participants with serious mental illness (N = 24) at a public specialized psychiatry hospital in South Africa and their caregivers (N = 6). We conducted an inductive thematic analysis with two pre-specified themes (risk factors for relapse and strategies for prevention), with the codes devised from the data.

**Results:**

Six sub-themes were identified from the analysis within the two pre-specified themes(Risk factors and strategies for relapse prevention): personal-related, family-related, and health system-related risk factors and strategies for preventing relapse, respectively. To highlight some essential findings, the importance of motivation for drug adherence, family involvement, and availability of anti-psychotic drugs in public health care were noted. More importantly, this study identified important cultural complexities where traditional healers play a significant role in some cultural understanding and treatment of mental illness, affecting medication adherence.

**Conclusion:**

This study calls for people-centered mental health care delivery in a public health system that listens to the voice of concern, including cultural challenges, and implements meaningful support that matters most to the patient and their family/caregivers.

## Introduction

Schizophrenia is a serious mental illness characterized by hallucinations, delusions, disorganized communication, poor planning, reduced motivation, and blunted affect [1]. Although the prevalence is estimated at 0.28% [2], schizophrenia is a debilitating type of serious mental illness that disrupts daily functioning [3]. It contributes to approximately 1.7% of the total years of a life lived with disability (YLDs) to the burden of disease globally [2]. Despite such challenges associated with schizophrenia, most individuals with schizophrenia can achieve long-term remission and functional recovery, provided appropriate care is secured [4]. However, many individuals living with schizophrenia experience relapses, with a considerable proportion of them being rehospitalized in specialized psychiatric hospitals [5, 6].

Schizophrenia is characterized by recurrent relapses, which contribute to the further downward drift in functioning, as the previous episodes increase the risk of further relapses [7]. A relapse is defined as the exacerbation or reemergence of positive symptoms accompanied by noticeable functional and behavioral changes after previous stabilization/remission [8]. Each relapse significantly burdens their caregivers and society and results in hospitalization, which leads to the chronic stage of the disease, cognitive impairment due to progressive structural brain damage, personal distress, incarceration, and interference with rehabilitation efforts [5, 8]. Relapse rates, often operationalized as psychiatric rehospitalization, vary from 50% to 92% in both developed and developing countries, despite the former having well-established mental health services [9]. The main risk factor for relapse is poor adherence to anti-psychotic medication, with others being substance use, comorbid psychiatric illness, medical and surgical conditions, stressful life events, and the treatment setting [5, 8, 10].

In managing schizophrenia and other forms of serious mental illness, psycho-social approaches, in combination with anti-psychotic drug therapy, may significantly reduce relapses compared to anti-psychotic treatment only [11, 12]. While the risk of relapse may be reduced with appropriate mental health services and optimum treatment, the extreme scarcity of mental health services (MHS) outside specialized psychiatric hospital settings are extensive across sub-Saharan Africa. In South Africa, MHS-related support is inadequate outside specialist public sector psychiatry hospital settings for individuals with severe mental illness (SMI). In KwaZulu-Natal (KZN) province, often considered the heart of the HIV endemic in South Africa, the rate of rehospitalization for schizophrenia is approximately 50%, with poor drug adherence being the main factor, this being patient-dependent [6, 13]. Engagement in care following discharge and linkages to community care is crucial. Only a few studies have explored the determinants of relapse in KZN, with little known about the strategies for its prevention from the perspective of individuals living with serious mental illness and their caregivers. In the absence of knowing the risk factors for relapse and strategies for its prevention from patients’ and caregivers’ perspectives, it will not be possible to address the recurrent relapse that is pervasive in individuals living with SMI in resource-limited settings.

## Methods

### Study setting and design

This study was conducted at a public specialized psychiatry hospital in Durban, South Africa. The qualitative approach used descriptive phenomenology with audio-recorded face-to-face interviews of inpatient study participants diagnosed with serious mental illness and their caregivers to explore their perspectives on the risk factors for relapse and strategies for its prevention. Specifically, we used the phenomenological approach compared to other qualitative methods to capture the shared meaning of the lived experience for patients and caregivers in the phenomenon of relapse [14, 15].

### Participants: Sampling and recruitment

The study participants were hospital inpatients with a history of psychiatric admissions who were in the recovery/treatment stage for two weeks and were selected using a consecutive sampling method. The additional inclusion criteria were 18 years or over, the ability to speak IsiZulu or English, and diagnosis with schizophrenia, schizoaffective disorder, and psychosis NOS, also known as other specified schizophrenic spectrum, using the Diagnostic and Statistical Manual 5^th^ edition. Patients unable to give informed consent (having more than 2 positive and negative symptoms) were not eligible. An independent clinical team at the psychiatry hospital (e.g., doctors, nurses, psychologists, social workers, pharmacists, and occupational therapists) assessed the capacity (not actively psychotic with 2 or fewer positive and negative symptoms) of the potential study participants to consent before they were referred to our study. As the study explores the risk factors for relapse and strategies for its prevention from caregivers’ perspectives, they were provided separate consent before their family members were approached to participate. The inclusion criteria for the above participant’s’ caregivers were 18 years and over, able to speak isiZulu or English, and living with a patient for more than six months, with written and separate consent for all study participants being obtained.

### Data collection

A trained interviewer, fluent in English and isiZulu, informed the 24 patients and six caregivers about the data collection procedure and conducted face-to-face interviews using a semi-structured interview guide. The core questions explored their experience of in-hospital mental health services, reasons for admission, medication types and adherence, reasons for missing medication, discharge plans, needs after discharge, reasons for relapse, and strategies for continuing care. The study participants received an honorarium of R50, with the caregiver receiving R150 to cover travel and meal costs. The interviews lasted approximately 60 minutes, with the data being collected from 10^th^ April 2019 to 24 July^th^ 2019.

### Data analysis

The demographic information was analyzed descriptively for both participants, while the qualitative responses were thematically analyzed. Each audio-recorded interview was transcribed verbatim before the following interview, with descriptive elemental coding occurring by repeated readings of the transcripts to develop initial codes [16]. The data analysis entailed using NVIVO 12 software and followed the inductive thematic analysis, the coding being data-driven [16–18]. To increase the trustworthiness of the data, three authors independently coded the text using NVIVO 12 software, after which they discussed any discrepancies and resolved through consensus, which resulted in the initial codebook being created. The clustered codes expressed the same or similar ideas to generate the sub-themes, with the relationship between the sub-themes being examined and matched with the two pre-specified themes.

### Trustworthiness

To ensure rigor in this study, four criteria of trustworthiness were observed (credibility, dependability, confirmability, and transferability). Credibility was ensured by obtaining a thick description of the phenomena: triangulating data from different sources (patients and caregivers), conducting member checking during data collection, asking the questions repetitively, and undertaking daily transcriptions to inform the following interviews. Dependability was ensured by using a specific interview guide for the patients and caregivers, conducting inter-coder analyses, and debriefing the initial results to the study team. Confirmability was ensured by keeping interview notes, audio recording the interviews, and doing verbatim transcriptions that enabled quotes to be presented. Lastly, a thickness description of the results was discussed with other studies in the same context to ensure transferability.

### Reflexivity

The interviewer reflected on the data collection process, kept daily notes, and did not interrupt the participants but sought clarity when necessary. The phenomena were described during the analysis/analyses, and the manifest, not latent, meaning from the data was presented.

### Ethics

Ethical approval was obtained with the reference number (BFC 189/17). Gatekeeper’s permission was obtained from hospital management, and all study participants received copies of their written informed consent form. To ensure confidentiality, participants were identified by number and not by name throughout the data collection and analysis process.

## Findings

### Demographic characteristics

Of the 24 inpatients interviewed, more than half were male (n = 14, 58%), the majority being Black African (n = 21, 88%), and the mean age 34.8 years (SD = 10.5 years). Half of the patients had incomplete secondary education (n = 12, 50%), the majority were not married (n = 17, 71%), and more than half had children (n = 13, 54%). Over three quarters (n = 19, 79%) were unemployed for six months before admission, and a third (n = 8, 33%) had experienced homelessness for six months before admission, with nearly half (n = 11, 46%) receiving care for drug abuse or alcohol. (Table 1). The majority of caregivers were Black African (n = 6, 100%) and female (n = 5, 83.), with a mean age of 47.6 (SD = 7.5), of whom 50% (n = 3) had secondary education (Table 2). The inpatients are indicated by PT (number) in the quotes, while the caregivers are indicated as such.

**Table 1:** Demographic characteristics of patients (N = 24).

Characteristics	N (%) or mean ± SD
**Gender**	
Male	14 (58.3)
Female	9 (37.5)
Other (Neither male nor female)	1 (4.2)
**Age**, mean± SD	34.8 ± 10.5
**Race**	
Black African	21 (87.5.)
Colored	3 (12.5)
**Education**	
Primary education incomplete	6 (25)
Primary education complete	3 (12.5)
Secondary education incomplete	12 (50)
Matric	1 (4.2)
Don’t Know (Failure to report education level)	2 (8.3)
**Marital status**	
Married	4 (16.7)
Don’t know (Failure to report marital status level)	3 (12.5)
Not married/single/widowed	17 (70.8)
Have child/ children	13 (54.2)
**Pre-treatment characteristics**	
Unemployed	19 (79.2)
Homeless or temporary housing	8 (33.3)
**Help received for abused drugs or alcohol in the past six months**	11 (45.8)

**Table 2:** Demographic characteristics of caregivers (N = 6).

Characteristics	N (%) mean ± SD
**Gender**	
Male	1 (17.7)
Female	5 (83.3)
**Age**, mean ± SD	47.6 mean ± SD 7.5
**Education**	
Secondary education	3 (50)
Incomplete Secondary	1 (16.7)
Incomplete primary	2 (33.3)
**Race (Black African)**	6 (100)

### Theme 1: Risk factors for relapse

The sub-themes that were inducted from the analysis were categorized into 1) personal, 2) family, and 3) health system-related risk factors, with the comments by the patients and their caregivers being presented together (Table 3).

**Table 3:** Thematic analysis of risk factors for relapse from the patient’s and caregivers’ perspectives (Theme 1).

Quotes	Participants	Codes	Sub-theme
*“I stopped taking it because it gave me too much diarrhea*.*”* *“Sometimes he defaults them because he feels like they make him gain weight*.*”*	Patient Caregiver	1. Poor anti-psychotic drug adherence	Personal-related risk factors
*“Not having transport money*.*”* *“She’s usually out of transport*.*”*	Patient Caregiver	2. Lack of transport	
*“I stopped using alcohol because I realized it was clashing with the pills*.*” “What he does is he smokes too much weed*.*”*	Patient Caregiver	3. Substance use.	
*“Because of my illness*, *I have lost my job*.*”* *“Secondly*, *when he does not have money*.*”*	Patient Caregiver	4. Lack of money	
*“I don’t know; perhaps she was still hurt by the passing of her mother*.*”*	Caregiver	5. Previous traumatic events	
*“I’d start with my aunt*, *who ended up saying that they don’t have money*.*”*	Patient	1. Lack of support	Family-related risk factors
*"Also*, *he didn’t have anyone to accompany him*.*”*	caregiver		
*“They look at me like I’m an incomplete man*.*”*	Patient	2. Lack of love and respect	
*“I think abuse and not receiving love*.*”*	Caregiver		
*“I’m talking about the personal problems he faces*.*”*	Caregiver	3. Family problems	
*“Yes*, *I believe in them because I went to one traditional healer*.*”* *“They made him barf and did incisions*, *and those things disturbed him mentally”*	Patient Caregiver	4. Family belief in traditional healers	
*“They write on the board ‘discharged*,*’ and you get fetched*.*”*	Patient	1. Lack of discharge plan and preparation	Health-system-related risk factors
*“It’s just that I haven’t been to the groups*.*”*	Patient	2. Lack of group therapy/support group	
*"Moreover*, *no one has ever given me an opportunity to sit down like this and ask about my worries*.*”*	Caregiver		
*“They discharged her when she was still the same*.*”*	Caregiver	3. Premature discharge	
*“Polly clinic lately*, *being out of medication*.*”*	Caregiver	4. Lack of drugs from clinics	

#### Sub-theme 1: Personal-related risk factors

Reported factors that increased the risk of relapse at the individual level were coded to create the sub-theme: poor anti-psychotic drug adherence, lack of transport, lack of money, substance use, and previous traumatic event.

*Code 1*. *Poor anti-psychotic drug adherence*. Most patients reported that the main reason for relapse was poor anti-psychotic drug adherence. The reasons were: forgetfulness, not being at home during medication time, and the feeling of recovery, with a consequent perceived lack of the need for medication. Factors relating to access, such as forgetting to carry their health card required to collect their medication, were indicated.

*"Maybe when I changed bags and didn’t carry the bag I was using, and maybe the green card is in the other bag. Then I end up forgetting and found the card another time"* (**PT 15, female 41 years)**

Fear of side effects was another issue, with some reporting weight gain, being sleepy, and having diarrhea, which interfered with their daily activities, especially when taking the medication in the morning before work.

*"Yes, I’ll be sleepy at work if I take my pills in the morning.” (* **PT 25, male 38 years)**

*"I stopped taking it because it was giving me diarrhea too much*. *After taking it over and over*, *I always had a hunger and a running stomach*." (**PT 23, female 42 years)**.

"*Sometimes he defaults them because he feels like they make him gain weight (***caregiver 5, female 33 years)**

The patients also mentioned that they did not always have food to eat, which made it difficult to take their medication as instructed.

*"Because I can’t drink pills on an empty stomach"* (**PT 13, male 36 years)**

*Code 2*: *Lack of transport*. Many referred to the lack of transport money to attend the follow-up clinic, which was often far from their homes and meant they could not access them on foot.

*"yeah! There was a problem with the distance, and the transport money wouldn’t be available when the pills finished*. (**PT 15, female age 41)**

*"It requires me to send her money for transport because she’s usually out of transport money*.*" (***Caregiver 3, male 47 years)**

*Code 3*: *Lack of money*. According to one caregiver, lack of money increases stress in the patient, causing him to become violent and demand everything in the family and increasing the risk for relapse.

*"When he does not have money, he becomes violent and wants money forcefully and demands everything in the house." (* **Caregiver 4, female 51 years)**

Also, a few patients mentioned that the illness led to them losing their jobs, and the loss of income increased the difficulty of catering to their needs, thereby increasing their aggression and the risk for relapse.

*"Also, about jobs, sister, we want jobs."(* **PT 6, Female 47)**

***“****yes*, *if I could get a job*. *I don’t want the white person’s job; I want the one where I’m going to sell for myself*. *I can sell a lot of things*, *or I could build a table with fruits and veggies and sit with sweets and everything*, *and I could sit down and sell just like that* **(PT 16 male 53 years***)*

*Code 4*: *Substance use*. Study participants noted that alcohol and weed/cannabis made patients forget to take their medication or adversely affected its effectiveness, as they underestimated the importance of using the prescribed medicine:

*"he does smoke too much weed."* (**Caregiver 4, female 51 years)**

*" I stopped using alcohol because I realized it was clashing with the pills*. *It mixes things up*, *you see; it’s such things*, *sister" (***PT 14, male 51 years)**.

*Code 5*: *Previous traumatic event*. One caregiver reported that their family member could not cope with a past bad experience and the associated negative emotion that was never dealt with, increasing the risk for relapse.

*"I don’t know, perhaps she was still hurt by the passing of her mother, and she hadn’t accepted that" (* **Caregiver 1, female 33 years).**

#### Sub-theme 2: Family-related risk factors

Reported family-related factors that increase the risk of relapse were coded as a lack of family support, love and respect, family problems, and family belief in traditional healers.

*Code 1*: *Lack of family support*. Most patients reported not having support from their family members, including being reminded about medication times, accompanying them during follow-up visits for drug collections, and providing money to cater to their needs, especially transport. One patient acknowledged that:

*"The first thing was money for transport, and sometimes you would find that I don’t have even a cent, then I had to beg my aunt and uncle. I’d start with my aunt, who said they don’t have money. Then I ended up begging my uncle. I know they don’t have money. They get a pension. I had to borrow from the neighbors, which created so many debts for me, and even worse, I wasn’t working anymore. How am I going to continue because I have to get my pills"?* (**PT 20, female 52 years).**

One caregiver added that some clients miss the appointment because there is no one at home to accompany them to the clinic:

*"Also, he didn’t have anyone to accompany him because his sister, the one he stays with at home, is not well. She is also sick. And maybe I’m here at work* ***." (*Caregiver 4, female 51 years)**

*Code 2*: *Lack of family love and respect*. One of the study participants reported relapsing due to anger caused by a lack of respect from the children around her home. However, the caregivers insisted that the lack of love from the family member creates anger in the patients, and the persistence of negative emotions increases the risk of relapse.

*"Also, tell the children not to undermine me, because that also affects my life, being bothered by* children” (**PT 15, female 42 years).**

*"I think abuse and not receiving love*. *Sometimes at her home*, *they wouldn’t pay attention to her*. *They locked her out and remained outside because they said she was fighting with them*. *But then*, *they also never thought of taking her to the hospital*. *"(***Caregiver 1, female 53 years)**

*Code 3*: *Family problems*. The caregivers mentioned that their family members experience recurring and unresolved challenges and that once discharged and returned to their unsupportive home environment due to life stressors, they continue to face these challenges, which results in relapse.

*"Her first problem was when social workers brought her back from XXX Hospital. She came that day to find out that her mother had passed away. I think that also took a toll on her mind" (* **Caregiver 2, female 53 years).**

*“I’m talking about the personal problems he faces; he has three children*, *two of them are in jail in Newcastle*, *and the other one*, *his daughter*, *has three kids*, *and she is dating a pastor*.*”* **(Caregiver 4, female 51 years).**

*Code 4*: *Family belief in traditional healers*. Few patients acknowledge that their families took them to traditional healers, which did not improve their condition. Furthermore, certain patients report that seeking treatment from traditional healers negatively impacts their adherence to prescribed medications and leads to additional financial expenses, whereas accessing medicines from the hospital is free:

*“My brother took me to the south coast to another traditional healer, who made me drink traditional tonics and said, “You must drink this one when you go to bed and vomit with this one; you will be healed.” I was not healed; the traditional medicines did not cure my mental illness. The hospital always cures me, and the hospital does it well, yes” (* **PT 17, male 44 years).**

*“I have been treated by traditional healers of different kinds*. *But they were not honest and said I know your pills; I can get them for you*. *I realized that my pills are essential*, *contrary to the treatment from traditional healers; meanwhile*, *accessing my pills is free of charge I don’t pay*, *so I’m wasting money”* **(PT 24, male 28 years).**

Also, one caregiver mentioned that they took their patient to traditional healers and hospitals at the same time. However, the patient spent a long time with a traditional healer, and they did not recognize any improvements:

*“He went to the traditional healer and the hospital at the same time. Because he resided with a traditional healer for a very long time, maybe close to a year, and there was no help. They made him barf and did incisions, and those things disturbed him mentally. The thing is, at home, we have different beliefs, but I live with him at home. honestly, I have given up on the side of traditional healers***”** (**Caregiver 5, female, 33 years).**

#### Sub-theme 3: Health system-related risk factors

The reported factors beyond the individual and family level that increase the risks of relapse were coded as the lack of a discharge plan and preparation, lack of group therapy/support group, premature discharge, and lack of drugs from the clinics.

*Code 1*: *Lack of discharge plan and preparation*. All patients reported being inadequately prepared for discharge, often did not receive instruction about how to handle their situation outside the hospital setting, and at discharge, they only received the pass-out without additional information.

*"They don’t help me with anything else. I do see others being discharged when their day has come. They write on the board ’discharged,’ and then you get fetched". (* **PT 8, male 28 years)**

*Code 2*: *Lack of group therapy/support group*. The patients mentioned that the hospital does not offer group support discussion, which is essential for them to share ideas on coping with their current situation and maintaining their daily medication intake.

*"but the group thing, I like it; I would be happy to do it because we share ideas, I would love that" (***PT 16, male 53 years)**.

One of the caregivers acknowledged not receiving any information on living and caring for family members with serious mental illness. In the absence of a support group, they could not express their worries and challenges and receive support.

*"Moreover, no one has ever given me an opportunity to sit down like this and ask about my worries and the challenges I face as a person who cares for someone with mental illness. I have never received an opportunity to be asked before since 2016 until 2019".* (**Caregiver 1, female 53 years).**

*Code 3*: *Premature discharge*. The caregivers mentioned premature discharge as a critical factor for rehospitalization, as they sometimes discharge patients who are unstable and should probably not be released.

*"When she came back from XXX, they discharged her when she was still the same; there was no change. She came back not sleeping and all that" (* **Caregiver 1, female 53 years).**

*Code 4*: *Lack of drugs from the clinics*. Caregivers shared the experience of not receiving the drugs from the clinics, being asked to return to collect the medications after a few days once the stock had arrived, and sometimes being given drugs with different names, which caused confusion.

"*If she runs out of medication and doesn’t collect it from here and goes to collect it at XXX, they say they’re unavailable; otherwise, she returns with a packet as big as this. But all of them are low dosages, and they don’t have similar names. If you dare to ask about the different names, it’s like, you have become a doctor yourself. And then you become quiet and leave it like that". (***Caregiver 3, male 47 years).**

### Theme 2: Strategies for preventing a relapse

The reported strategies for relapse prevention were also categorized into personal, family, and health system-related factors, with the comments by the patients and their caregivers being presented together (Table 4).

**Table 4:** Thematic analysis of the strategies for relapse prevention from patients and caregivers’ perspectives (Theme 2).

Quotes	Participants	Codes	Sub-theme
*“I should collect medication all the time*.*”*		1. Self-Motivation for drug adherence	Personal-related strategies
*“I need to have a partner*.*”*		2. Need for a partner	
*“Working*, *getting a place to live*.*”*	Patient	3. Having a job	
*“I am going to school*.*”* *“Continuing to go to church*.*”* *“I need to drop the weed completely*.*”*		4 Continue with education, religious activities, and stopping the substance use.	

*“What bothers me most are the daytime activities*.*”*	Caregiver	5. Daytime activities	
*“My family to be given my date always*.*”* *“Always tell her we should collect her medication and the clinic*.*”*	Patient Caregiver	1. Family involvement	Family-related strategies
“I have asked the hospital to help me apply for ** to get the government grant.*”*	Caregiver	2. Follow-up with grant money	
*“I have given up on the side of traditional healers”*	Caregiver	3. Focus on hospital treatment only	
*“Yes*, *you can bring the social worker*.*”*	Patient	1. Need for a social worker	Health system-related strategies
*“Yeah*! *There was a problem with the distance*	Patient	2. Using the nearest clinic	
*“It would be better if the clinic wasn’t too far*.*”*	Caregiver		
*“When you form your group and discuss*.*”*	Patient	3. support group/group therapy	
*“Yes*, *I would love to be part of the support group*.*”*	Caregiver		
*‘You can help by writing it down*, *so I could see*.*”*	Patient	4. Filling the card with the correct information	
*“Make him get a 1-monthly supply injection until the next appointment again*.*”*	Caregiver	5. Monthly Injection	

#### Sub-theme 1: Personal-related strategies for preventing a relapse

The reported strategies for relapse prevention at the individual level were categorized into the codes of self-motivation for drug adherence, the need for a partner, having a job, continuing with education, religious activities, and stopping substance use and daytime activities.

*Code 1*: *Self-motivation for drug adherence*. Most patients planned to adhere to treatment and were motivated by the positive outcome, as one patient claimed:

"*I should collect medication all the time. Because if I happen to be angry and I take them, the anger subsides; that’s why I think I should take them". (***PT 17, male 44 years).**

*Code 2*: *Need for a partner*. Some patients said they needed a partner who would show them love and care and remind them of the time to take their pills:

*“It’s just that I need a partner to stay with me that would be helpful to me.*” (**PT 11, male, 30 years).**

*Code 3*: *Having a job*. A few patients said they would look for a job that would prevent them from being idle and associating with bad people and that the money earned would help to meet various needs:

*“Yes, I was thinking of working and fixing the gearbox. After fixing the gearbox, I’ll get paid and save some money, and perhaps buy a house and find my proper place, and then buy a car that I am going to travel with”* (**PT 13, Male 35**)

*Code 4*: *Continue with education*, *religious activities*, *and stop substance use*. A few patients said that after discharge, they would continue their schooling, participate in religious activities, and stop consuming alcohol and smoking cannabis.

*“What would be helpful for me is continuing to go to church and preaching the gospel because I am a Christian. And continue with God’s values and have a good spirit, not a bad one, because I don’t have an evil spirit*.” **(PT 9, male 31 years)**.

*"When I’m discharged from here*, *I do not want to touch cigarettes*, *and I don’t want touch weed*.*" (***PT 17, male 44 years).**

*“I am going to school*: *at school*, *I do physics*, *business economics*, *and geography***” (PT4, Female 25 years)**

*Code 5*: *Daytime activities*. Daytime activities were also a concern for all caregivers, as their family members were not employed or keeping busy, which resulted in their associating with undesirable people. The caregivers felt that doing something constructive during the day would help the patient develop new skills, increase self-confidence and social support network, and improve quality of life:

"*What bothers me most are the daytime activities because he is not doing anything during the day. This also affects his self-esteem because he’s not doing anything. His biggest wish is to do something. So, I also think that amongst all his frustrations and stresses, should he get something that will keep him busy, things will be much better*." (**Caregiver 5, female, 33 years)**.

#### Sub-theme 2: Family-related strategies for preventing relapse

Family-related strategies to prevent relapse at the family level were coded as family involvement, helping the patients follow-up with the grant money and focus on hospital treatment only.

*Code 1*: *Family involvement*. Most participants expressed the need for family engagement as part of their support to continue with outpatient care.

“*By having someone from home who will be taught at least one, or my family, to be given my date always because my card is always with them. So they could be told to always remind me about my dates”. (***PT 9, male 31 years)**

The caregiver insisted that they play important roles in helping the patient to continue with the care, with one narrating that they will make sure that they fulfill the needs of the patients regarding attending the follow-up outpatient visits.

*"Always I tell her that we should go and collect her medication at the clinic because she loves school, so she can go back to school." (* **Caregiver 1, female 53 years)**

*Code 2*: *Follow-up with the grant money*. The caregivers identified the need to address issues related to the grant money so that their family members could have the means to attend their follow-up clinic visits.

*"I have asked the hospital to help me apply for him to get the government grant. This grant will help us have a transport fare for when we collect his medication when he’s back home. It was better when he was still here in the hospital because I was the only one who took a taxi to come and visit him. Now he is at home, and we both need money when we come for his appointments or to collect the medication. The doctors said we should bring our IDs the next time we come, but today we forgot. We’ll bring them on the 26th when we come to collect medication." (* **Caregiver 2, female 53 years)**

*Code*: *3 Focus on hospital treatment only*. Few caregivers acknowledge stopping taking their patients to traditional healers as they see no improvements in their patients. Instead, they prioritize hospital treatment and direct their attention solely toward it:

*“let me just put it this way; we’ve made peace with the fact that traditional healers are not helping him. So, we’re focusing on the health side of things, but before that, we took him pretty much everywhere”.* (**Caregiver 5, female, 33 years)**

*There is belief*, *but since he is getting from this hospital*, *we prefer to keep him receiving help from the doctors*. *Also*, *because it is free of charge and works for him*, *whereas in traditional healers*, *you have to pay*, *some of their tonics are harsh*. *And maybe he has to steam*, *use an enema to detox*, *and vomit*, *some of these things are dangerous*. **(Caregiver 4, female 51 years)**

### Subtheme 3: Health system-related strategies for preventing relapse

The strategies for preventing relapse beyond the personal and family level were coded as the need for a social worker and support/ therapy group, the availability of medication for collection from the nearest pick-up point, having the correct information on the card, and being able to have a monthly injection:

*Code 1*: *Need for a social worker*. The need for a social worker who they could access was expressed by most patients, particularly if they planned and visited the family member in their homes to explain how they should live with those on medication both before they came home and after they had been discharged.

“*I would love that Maybe if I am also there, to speak to them when I am also there, to explain to them so they would see. And for them to speak out whatever is inside them about what is troubling them"* (**PT 9, male 32 years)**

*Code2*: *Using the nearest clinic*. Several respondents indicated that collecting the medication from the nearest primary health care clinic would be useful to reduce the cost associated with long trips to a specific outpatient clinic at a hospital. A caregiver said attending clinics near their home was essential as they would not require money for transport, and the government should ensure that the drugs are always available at all clinics for collection.

*“My mother is complaining about the costs of coming here." (* **PT 22, male 24 years)**

*"It would be better if the clinic wasn’t too far*.*" (***Caregiver 1, female 53 years).**

*Code 3*: *Support group/group therapy*. Caregivers and patients indicated a need for support groups to discuss and share what they are going through and to better cope with their situation.

*"Yes, I would love to be part of such groups because it would also help me with answers, to gain more knowledge on how to treat my child, especially when he speaks something that does not make sense." (* **Caregiver 2, female 53 years).**

*“When you form your group and discuss what is upsetting each person*, *and then you give them help*, *you support them*.*”* **(PT 5, male 24 years)**

*Code 4*: *Fill the card with the correct information*. Most participants explained the importance of healthcare providers writing the correct follow-up visit date on the health card, as they rely on the data for keeping their appointments.

*"I always check the card for when I should return, and my family needs to be given my date always." (* **PT 12, female 19 years)**

*Code 5*: *Monthly injection*. The caregiver suggests that an alternative to the daily drug intake would be a monthly injection to help them stay well without having a daily drug intake.

*"I would make him not drink pills daily because he’s unavailable sometimes. I would make him get a 1-monthly supply injection until the next appointment again." (* **Caregiver 5, female 33 years).**

## Discussion

The current study explored the risk factors associated with relapse and identified strategies to prevent relapse among individuals with serious mental illness. Although there are some overlap across the sub-themes, we discuss the major findings from the reported personal-related, family-related, and health system-related factor sub-themes that are associated with relapse risks, with an emphasis on strategies in this section.

### Sub-theme 1: Personal-related risks and strategies factors

In this study, the patients and caregivers mentioned poor anti-psychotic drug adherence as a risk factor for relapse, consistent with previous studies [5, 8, 19]. Lack of transport money to attend the follow-up visits for drug refills was the reason for poor drug adherence, as participants from this study reported living a long distance from where they should collect their drugs. Furthermore, anti-psychotic medications are associated with side effects that make a few patients stop taking them, while some defaulted due to their experiences of gaining weight and having diarrhea, similar to Geddes [20]. A few patients reported forgetting the time for taking medication and that they did not have the food needed to enable their pill-taking, as noted by a number of authors [5, 8, 19]. To improve medication adherence, healthcare providers should provide comprehensive information about the potential side effects of anti-psychotic medication, including when to seek medical attention if they experience them. Additionally, automated phone calls and text messages can be implemented as a reminder system to ensure patients take their medication as prescribed.

However, the study patients are motivated to continue using the anti-psychotic medication as they perceived its benefit as enabling them to sleep well, with positive attitudes toward the benefits of anti-psychotic medication having been reported to increase adherence [21]. While some patients experienced side effects from the medication, the perceived benefits outweighed the perceived threat of psychosis symptoms, as supported by the Health Belief Model theory [22]. This highlights the need for incorporating teaching about the value of the anti-psychotic drug into the treatment plan to address modifiable behaviors for ensuring ongoing self-motivation and improved adherence.

Other patients understood the need to taking responsibility for their mental health and wanted to continue engaging in religious activities to help adhere to their treatment and prevent a relapse. While some religious teachings encourage a ’miracle cure’ and discourage the use of anti-psychotic medication [23], others provide the support that enables them to cope with schizophrenia. Being religious often increases the patient’s ability to cope and adhere to treatment and improves the quality of life due to strong social and spiritual support [5]. Health professional workers should create awareness of the importance of anti-psychotic medication among religious leaders and thereby create an additional support system for patients, as religion and spirituality can be important for people suffering from schizophrenia.

Some study participants also acknowledged using cannabis and alcohol, which resulted in them defaulting from taking medication as they forgot the time, needed to take it, and did not see the importance of taking it. Various studies consistently show the risk of substance use and psychosis [5, 8]. This is common for schizophrenia and can lead to relapse, independent of the other factors [24]. Some study patients acknowledged stopping smoking weed and involving themselves in productive activities, which helped to reduce the likelihood of substance use, e.g., looking for a job. In addition, caregivers noted the need for daytime activities for patients. Keeping busy with familiar tasks gives a sense of purpose and achievement that can give meaning to life and prevent substance use. An inability to perform daily tasks is likely to cause further distress to patients with severe mental health problems [25], with a stable daily routine linked to feelings of well-being among patients with schizophrenia [26]. The social learning theory postulates that behavior can be learned through observation, imitation, and reinforcement [27]. Daily activities provide opportunities for individuals to observe and learn healthy behaviors and an alternative source of reinforcement, which can decrease the likelihood of turning to substance use. The occupational therapist should consider building vocational skills for patients, as improved functionality

### Sub-theme 2: Family related-risk and strategy factors

In this study, a few participants reported relapsing due to criticism from their family members, while some missed their follow-up visits as no-one could accompany them, and sometimes lacked travel money as their families could not support them. Other patients claimed the problems they face in their families increase the risk of relapse, such as the lack of family love and support [5, 28, 29]. The patients and caregivers suggested the involvement of family members as a strategy for relapse prevention by accompanying them for follow-up visits and monitoring medication intake, with the advantages of family motivation and support on the outcome of schizophrenia having been widely recognized [5, 30]. The family’s involvement increases adherence to treatment and reduces relapse, with a supportive family expressing acceptance, warmth, understanding, and encouragement, which are predictors of recovery [12, 28, 31]. Health professional workers should involve family psychoeducation as part of the treatment, which will help improve family engagement.

Other patients wanted to build strong partner relationships as they felt they would remind them of the time and motivate them to take their medication, thereby increasing adherence and reducing the risk of relapse. Studies show that support from partners, including assisting with medication reminders and accompanying them for follow-up visits, reduces the risks of relapse [32, 33]. The evidence from an HIV study shows that patients living with a partner are more likely to adhere to treatment [34]. People with serious mental illness are said to lack interest in connecting to others, highlighting the need for health professional workers to involve family psychoeducation in treatment to build/establish social relationships and promote social skills training.

Moreover, patients and caregivers have recognized that their trust in traditional healers impacts their adherence to anti-psychotic medication, leading to increased costs compared to hospital treatment. Instead, caregivers have decided to prioritize hospital treatment over seeking assistance from traditional healers. In low-income countries, traditional healers are highly regarded and recognized for treating schizophrenia [35–39]. Healthcare providers should educate family members on the significance of medication adherence and the advantages of modern treatment compared to traditional approaches.

### Sub-theme 3: Health system-related risks and strategies factors

In this study, the patients and caregivers highlighted that the lack of group therapy/support group, discharge plans, and preparation prevented them from being ready to cope with the adverse after being discharged due to a lack of problem-solving skills. The caregivers, in particular, felt ill-equipped to take on the responsibilities associated with caring for affected persons. Access to people who could guide them due to their own experiences would be invaluable. The patients suggested the involvement of a social worker for group counseling and family therapy to reduce the risk of relapse and provide a support system for those caring for affected persons. Social workers are an integral part of psychotherapy treatment, and incorporating them into practice will be useful in successfully transitioning patients from inpatient hospital care to outpatient support. The lack of social workers and clinical psychologists in SA is a pressing matter, particularly in underprivileged areas with limited mental health services. To overcome this challenge, task shifting is a potential solution. This strategy involves training non-specialists, such as community health workers or nurses, to deliver mental health care, including psychoeducation, within the community. By doing so, more individuals needing mental health services can receive care, even if a shortage of specialized professionals is available.

Psycho-social education is critical for enhancing the patient’s overall function, quality of life, motivation, and adherence to prescribed treatment [1, 11]. However, the evidence shows that patients with schizophrenia are usually discharged from hospitals to follow-up at their nearest community mental health clinics, focusing mainly on pharmacotherapy [40] and not educating patients and caregivers on managing a complex health condition. Ensuring enough healthcare workers trained to provide psychoeducation can be challenging in healthcare settings. Additionally, it’s important to consider patients’ factors, such as cognitive ability and literacy level, as these can significantly impact the effectiveness of psychoeducation. However, using simplified language and providing psychoeducation to patients and caregivers may be more effective in addressing these challenges.

Engagement in care after hospital discharge is essential for patients with schizophrenia [41], with preparation for their return to their communities being critical. The evidence indicates that the transition to the community and continuing with follow-up care is affected by the lack of unmet needs and failure to cope with their daily life, which increases the risk of relapse [6]. In addition to lack of post discharge planning and provision of resources, the patients and caregivers also acknowledged that early discharge before remission and shortage of medications from the clinics increased the risks for relapse, consistent with the findings by [42]. This indicates the need for caregivers to be involved in discussions about discharge dates to avoid situations detrimental to the patient’s health, increasing the likelihood of relapse and readmission. Caregivers need to be informed about the conditions under which the patient would be released, what changes had occurred, how they could be expected to behave on discharge, and under what circumstances they need to be reevaluated for readmission.

In addition, the nearby availability of anti-psychotic drugs is one of the challenges reported in this study, as indicated by [19]. While medical services in SA are free at public sector facilities, the accessibility of anti-psychotic drugs is a barrier to optimum mental health services. Some second-generation antipsychotic drugs may only be available in specialized public sector psychiatry outpatient clinics or regional hospitals [8]. Ensuring a regular and adequate supply of appropriate, safe, and affordable medication is one of the challenges in public sector mental health services [11, 12, 19, 43]. The inconsistency in drug availability and accessibility and lack of consistency in the names of the medication provided (generics) need to be addressed to reduce confusion and relapse. Given the challenges associated with ongoing medication uptake, caregivers suggested using long-acting -injectables rather than taking a daily pill, as adherence to anti-psychotic drugs is the mainstay of preventing relapse in schizophrenia. Using atypical drugs with fewer side effects has advantages over ‘typical’ drugs with more extra-pyramidal side effects. Studies show that most patients prefer long-acting injectable (LAI) formulae of anti-psychotics over oral medication, which obviates the need to remember to take medication daily [20, 42]., In addition, the use of the public sector central chronic medicine dispensing and distribution (CCMDD) program, which allows patients to collect medication nearer to home, may be used to improve access to patients with schizophrenia by allowing the patients to pick up the medication at their convenience place. The effectiveness of the use of CCMDD in improving access has been observed in chronic diseases such as HIV. However, LAI is not available on CCMDD; hence the availability and accessibility of anti-psychotic drugs should be ensured at all levels of health care provision, thus obviating the need for people with limited resources to travel long distances every month to collect medication.

### Study limitation

We acknowledged that the inclusion of inpatients soon after relapse could have impacted their reflective capacity. The caregiver sample size was small relative to the patient sample size, determined by those who agreed to participate. Future studies may need to engage family members and caregivers and survey a community sample of people with schizophrenia. Additionally, the findings describe the patients’ perspectives on the barriers and facilitators around transition and do not fully capture the actual practice after discharge from the hospital.

### Future directions

This study summarized the strategy for preventing relapse among patients with schizophrenia from the patients’ and caregivers’ perspectives, with both identifying the importance of self-motivation and personal responsibility for drug adherence. This needs to be supported by healthcare and social workers who ensure that patients develop positive attitudes toward medication during psychoeducation sessions and by incorporating family intervention therapy to increase their involvement in the treatment. Adequately preparing patients and their caregivers to be able to cope after discharge, providing access to support services as well as being able to access mental health care services closer to those who need them is also essential. A holistic approach to family, caregivers, and patient support is essential for reducing the mental health treatment gap that exists in South Africa and preventing relapses. In addition, a quantitative study with involvement of community members is warranted with further exploration of additional risk factors and preventive measures for relapse.
